# Course of Pregnancies and Occurrence of Acute Pancreatitis in Women With Chylomicronemia

**DOI:** 10.1210/clinem/dgaf409

**Published:** 2025-07-15

**Authors:** Miriam Larouche, Jean Bergeron, Diane Brisson, Nathalie Laflamme, Noémie Audet-Verreault, Claire Sharon, Sybil Charrière, Melanie Rama, Delphine Collin-Chavagnac, Philippe Moulin, Daniel Gaudet

**Affiliations:** Department of Medicine, Université de Montréal and ECOGENE-21, Chicoutimi, Canada G7H 7K9; Department of Specialized Medicine and Endocrinology and Nephrology Unit, CHU de Québec, Université Laval and CHU de Québec—Université Laval Research Center, Québec City, QC, Canada G1V 4G2; Department of Medicine, Université de Montréal and ECOGENE-21, Chicoutimi, Canada G7H 7K9; Endocrinology and Nephrology Unit, CHU de Québec—Université Laval Research Center, Québec City, QC, Canada G1V 4G2; Department of Medicine, Université de Montréal and ECOGENE-21, Chicoutimi, Canada G7H 7K9; Department of Endocrinology, Louis Pradel Hospital, Hospices Civils de Lyon, Bron 69500, France; Department of Endocrinology, Louis Pradel Hospital, Hospices Civils de Lyon, Bron 69500, France; CarMeN Laboratory, UMR INSERM U1060, Oullins 69921, France; Department of Biochemistry and Molecular Biology, HCL, Pierre-Bénite 69495, France; CarMeN Laboratory, UMR INSERM U1060, Oullins 69921, France; Department of Biochemistry and Molecular Biology, HCL, Pierre-Bénite 69495, France; Department of Endocrinology, Louis Pradel Hospital, Hospices Civils de Lyon, Bron 69500, France; CarMeN Laboratory, UMR INSERM U1060, Oullins 69921, France; Department of Medicine, Université de Montréal and ECOGENE-21, Chicoutimi, Canada G7H 7K9

**Keywords:** pregnancy, acute pancreatitis, persistent chylomicronemia, LPL deficiency, familial chylomicronemia syndrome, multifactorial chylomicronemia syndrome

## Abstract

**Objective:**

Chylomicronemia is characterized by extreme hypertriglyceridemia (triglyceride values >10 mmol/L). It may be caused by a biallelic combination of a pathogenic variant [familial chylomicronemia syndrome (FCS)] or by genetic susceptibility combined with comorbidities and environmental factors [multifactorial chylomicronemia syndrome (MCS)]. Acute pancreatitis (AP) is the most serious complication of chylomicronemia. In the general population, the prevalence of AP during pregnancy is estimated to be <0.35%. As triglyceride levels significantly increase during pregnancy, it may affect the course of pregnancy and further increase the risk of AP in women with chylomicronemia.

**Methods:**

One hundred sixteen pregnancies involving 49 European and North American women with a history of chylomicronemia (20 FCS, 29 MCS) were retrospectively reviewed. The occurrence of AP, the course of pregnancy, fetal development, and delivery were evaluated.

**Results:**

Forty-two percent of FCS and 10% of MCS women experienced at least 1 AP episode during pregnancy (*P* = .01). Compared to MCS, women with FCS presented a higher percentage of pregnancies with AP (17% vs 5%, *P* = .02). Among all reviewed pregnancy-related AP, 56% occurred in primigravida FCS women compared to 0% in MCS. Premature deliveries were elevated in both groups, although they were more frequent in FCS (56%) vs MCS (19%) (*P* = .01). The percentages of miscarriages (11.8% vs 10.7%) and fetal failure to thrive (5.9% vs 9.2%) were not significantly different between the 2 cohorts.

**Conclusion:**

In this study, pregnant women with chylomicronemia had a 30-fold (MCS) to 120-fold (FCS) higher occurrence of AP compared to the general population. Chylomicronemia per se does not seem to influence fetal development.

Chylomicronemia is characterized by plasma triglyceride (TG) levels above 10 mmol/L (880 mg/dL) and is most often caused by a combination of genetic and environmental factors and/or comorbidities (1). Chylomicronemia can be sporadic, intermittent, or persistent (2, 3). Chylomicronemia is persistent when it occurs in more than half of TG measurements despite treating secondary causes and using lipid-lowering therapies (2). The genetic form of persistent chylomicronemia, called familial chylomicronemia syndrome (FCS), is a rare mendelian autosomal recessive disorder caused by a biallelic combination of pathogenic variants in the lipoprotein lipase (LPL) gene or the LPL machinery (*APOA5, APOC2, GPIHBP1, LMF1, CREB3L3*) (4, 5) leading to LPL deficiency, sustained accumulation of chylomicrons in the bloodstream and persistent severe hypertriglyceridemia (sHTG) (4, 5). However, a subset of patients with an elevated FCS score (6, 7) presents the FCS phenotype without necessarily carrying a combination of documented pathogenic variants (clinical FCS) (2, 3, 8). On the other hand, multifactorial chylomicronemia syndrome (MCS) is most often polygenic, combining genetic susceptibility and secondary factors such as type 2 diabetes, obesity, partial or generalized lipodystrophies, pharmacological agents affecting TG levels, and inappropriate life habits (9). Unlike FCS patients, those affected by MCS usually present a larger variability in TG levels. These patients accumulate both chylomicrons and remnants from the exogenous pathway as well as TG-rich lipoproteins from the endogenous pathway [very low density lipoproteins (VLDL) and intermediate density lipoproteins] (10). The severity of the MCS phenotype depends at least in part on the genetic susceptibility that includes heterozygous carriers of pathogenic or loss-of-function variants in genes involved in lipase-mediated vascular TG hydrolysis (11).

The main risk associated with chylomicronemia is acute pancreatitis (AP), which can lead to temporary or permanent organ failure and is associated with high mortality rates (12). Depending on regions, hypertriglyceridemia (HTG) is the third or fourth cause of AP globally (13), and patients with chylomicronemia have a high prevalence of recurrent AP (10).

Pregnancy is characterized by several modifications in lipid regulation, mostly due to hormone changes. Plasma TG concentration increases during the second and especially the third trimester, driven by the increase in estrogens and placental lactogen, 2 hormones that substantially decrease LPL activity during pregnancy (14-16). It has been demonstrated that, taken together, these 2 hormones might decrease LPL activity by up to 85%. Moreover, estrogen elevations are associated with an increased VLDL production by the liver. Consequently, plasma TG levels may increase by 2.5-fold at the end of pregnancy (17). Patients with chylomicronemia are thus at higher risk of AP during pregnancy. The prevalence of idiopathic AP during pregnancy in the general population is estimated to be less than 0.35% (12, 18, 19). It is documented that the risk of AP is elevated in the presence of sHTG, particularly among FCS, but less is known regarding the risk of HTG-related AP in pregnant women with recurrent or persistent chylomicronemia of any cause (8). The aim of this study was to document and compare pregnancies in women with sHTG affected by FCS or MCS, with a special emphasis on AP risk.

## Methods

Pregnancies of women with chylomicronemia were retrospectively reviewed in 3 lipid clinics in Canada and France (Chicoutimi, Canada; Québec City, Canada; Lyon, France). Data collection included questionnaires and a review of medical records. All participants presented with at least 1 episode of plasma TG concentration above 10 mmol/L (880 mg/dL). The severity of chylomicronemia was classified as persistent (more than 50% of plasma samples with TG concentrations >10 mmol/L) or not (3, 8). FCS diagnosis was established by the identification of biallelic pathogenic FCS-causing variants in the *LPL* gene or co-factors (*APOA5, APOC2, GPIHBP1, LMF1, CREB3L3*) or in the presence of an elevated FCS clinical diagnosis score (6, 20). Women carrying at least 1 pathogenic or otherwise loss-of-function variant in the *LPL* gene (21, 22) or co-factors and/or additional susceptibility factors such as obesity and type 2 diabetes were included in the MCS group. The occurrence of AP, the course of pregnancy and delivery, fetal development, follow-up of pregnancies, triglycerides monitoring, use of lipid-lowering drugs, or use of preventive plasmapheresis when appropriate have been retrospectively evaluated from the available data in the medical file. The diagnosis of AP was established based on the revised Atlanta diagnosis criteria (23) as previously described (24). Data comparisons were made using chi-square, Fisher's exact test, Student's *t*-tests, and ANOVA for independent samples. Statistical analyses were performed using SPSS package version 25 (IBM Corp., Armonk, NY, USA). This study (reference protocols: ECO HyperTG-Hyperchol in Canada and GENELIP/ASAP in France) was conducted in accordance with the principles of the Declaration of Helsinki and the Good Clinical Practice guidelines of the International Conference on Harmonization. Ethical approval was obtained from the Advarra institutional review board (last renewal in 2024) in Canada and from CNIL approval N°920434 and research project no. 05-07-06 approved by the Comité de Protection des Personnes in Biomedical Research, Paris, Necker in France.

## Results

Patients’ characteristics are presented in Table 1. Briefly, a total of 116 pregnancies involving 20 FCS women (51 pregnancies) and 29 MCS women (65 pregnancies) were reviewed. Both groups were comparable for age during pregnancies, body mass index, maximum documented TG levels, occurrence of gestational diabetes, and comorbidities. The diagnosis of chylomicronemia was already known prior to the first pregnancy in 73% of FCS but in only 20% of MCS women, respectively (*P* < .00001). Five patients were pharmacologically treated with a conventional TG-lowering agent during their pregnancy—most with omega-3. Although not significant, a higher percentage of FCS women tended to have documented AP episodes outside pregnancy and at a younger age compared to MCS.

**Table 1.** Patients’ characteristics

Variables	FCS (n = 20) (51 pregnancies)	MCS (n = 29) (65 pregnancies)	*P*-value
Genetic background (LoF variants)			<.00001
HoLPL	12	0	
HeLPL	0	11	
Compound LPL	4	2	
HeAPOA5	0	2	
Digenic	4	11	
Unknown	0	3	
Number of pregnancies per women	2.6	2.2	NS
History of AP outside pregnancy (%)	65	38	.074
Age at first AP episode (mean ± SD)	24.3 ± 7.4	42.3 ± 6.5	<.00001
Number of pregnancies with previously known diagnosis of chylomicronemia, n (%)	37 (73)	13 (20)	<.00001
Age at first pregnancy (mean ± SD)	26.0 ± 4.7	26.1 ± 5.0	NS
Age at last pregnancy (mean ± SD) all	29.6 ± 4.5	31.4 ± 4.6	NS
BMI before first pregnancy (mean ± SD)	21.8 ± 3.1	23.5 ± 5.0	NS
Obesity (%)	5	9	NS
History of gestational diabetes (%)	32	29	NS
Maximum documented TG levels (mmol/L) (mean ± SD)	50.2 ± 22.8	45.6 ± 29.7	NS
Use of LLD during pregnancy*^a^* (%)	19%	4%	.1
Use of MCT oil (%)	31	0	<.00001

Obesity is defined as BMI ≥ 30 kg/m^2^.

Abbreviations: AP, acute pancreatitis; BMI, body mass index; FCS, familial chylomicronemia syndrome (biallelic carriers and clinical FCS); He, heterozygote; Ho, homozygote; LLD, lipid-lowering drugs; LoF, loss-of-function; LPL, lipoprotein lipase; MCS, multifactorial chylomicronemia syndrome (all subtypes); MCT, medium-chain triglycerides; NS, nonsignificant (*P* > .1); TG, triglycerides.

^*a*^LLD included omega-3 or fenofibrate (1 FCS patient). One patient received plozasiran (APOC3 siRNA) 2 months before conception.

### Acute Pancreatitis and Pregnancies

**Figure 1.**
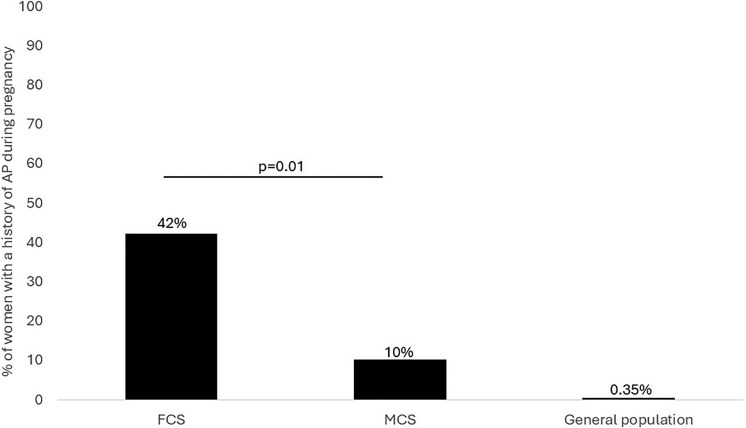
Percentage of FCS and MCS women with a history of AP during pregnancy. A higher proportion of women with FCS experienced at least 1 episode of AP during pregnancy compared to MCS. However, both groups present a higher prevalence of AP during pregnancy than the estimated prevalence in the general population (0.35%) (12, 18, 19). Abbreviations: AP, acute pancreatitis; FCS, familial chylomicronemia syndrome; MCS, multifactorial chylomicronemia syndrome.

A larger proportion of FCS than MCS women experienced at least 1 episode of AP during pregnancy (42% vs 10%, *P* = .01) (Fig. 1). This proportion was not significantly different when dividing FCS into biallelic carriers of FCS causing pathogenic variants or clinical FCS (data not shown). The diagnosis of chylomicronemia was revealed by elevated TG levels (>10 mmol/L) during the pregnancy in 5 FCS (25%) and 5 MCS (17%) women. Among them, the diagnosis was established following an AP episode in 2 FCS and 3 MCS women, respectively.

**Figure 2.**
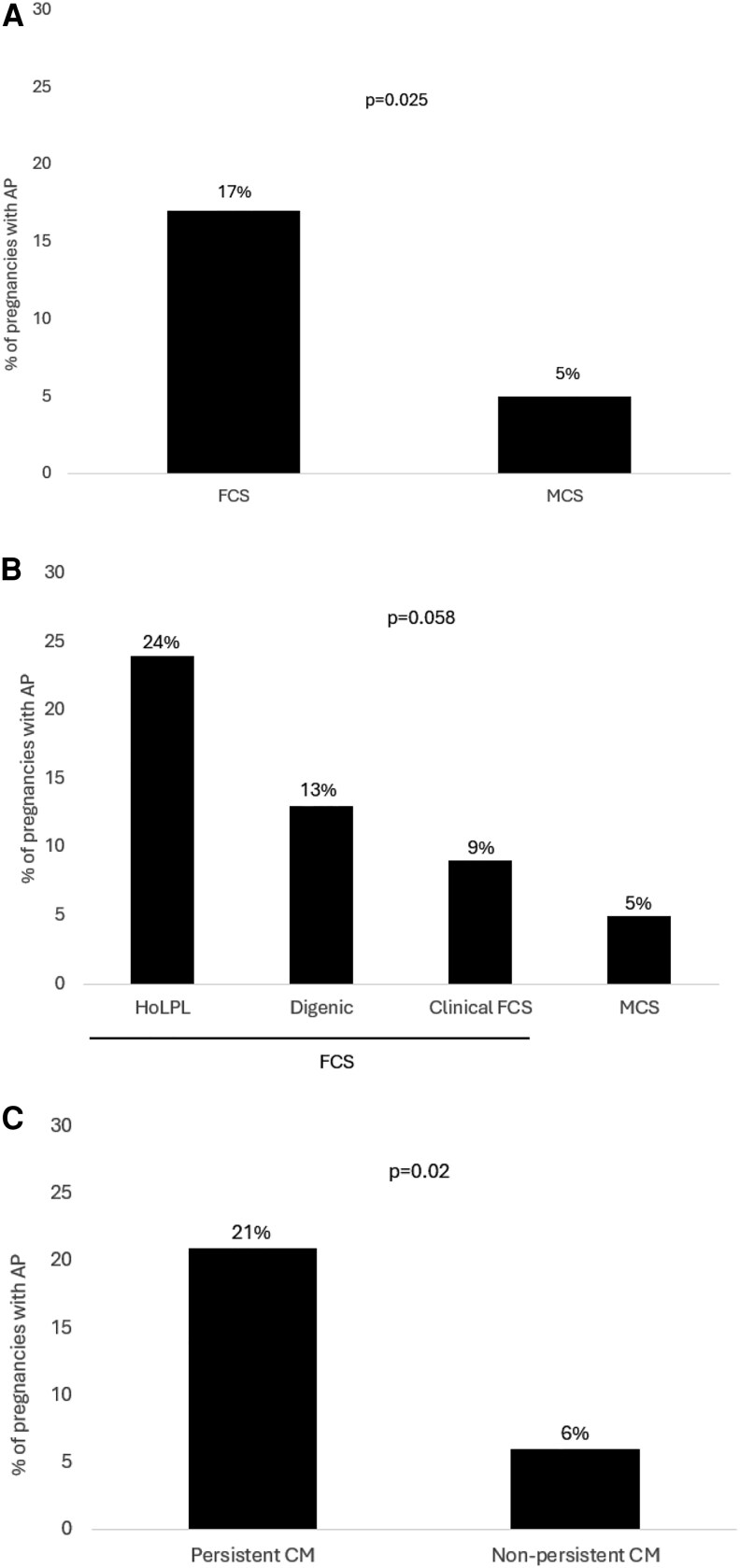
Percentage of pregnancies with AP in FCS and MCS women. (A) The proportion of pregnancies with AP was 3-fold more frequent in FCS (17%) than in MCS (5%). When stratified per genotype (B) or persistency of chylomicronemia (C), pregnant women carrying a biallelic combination of a pathogenic variant in the *LPL* gene or with persistent chylomicronemia of any cause tended to have more AP. Abbreviations: AP, acute pancreatitis; CM, chylomicronemia; FCS, familial chylomicronemia syndrome; HoLPL, carriers of biallelic combination of pathogenic variant in the LPL gene; MCS, multifactorial chylomicronemia syndrome.

Overall, FCS women presented a significantly higher percentage of pregnancies with AP than MCS (17% vs 5%, *P* = .02) (Fig. 2A). This trend was still observed when patients were classified according to their genotype (Fig. 2B) or the persistence of chylomicronemia (Fig. 2C). Among all reviewed pregnancy-related AP, 56% occurred in primigravida FCS women, whereas none occurred during the first pregnancy in MCS.

FCS women benefitting from a multidisciplinary (including nutritional) follow-up during pregnancies had more than 5-fold fewer AP episodes than the others (7% vs 39%, *P* = .01). A similar trend was observed in the 65 pregnancies in the MCS group as no AP episode occurred in the 4 pregnancies that were monitored compared to 3 episodes in the 61 pregnancies not followed by a multidisciplinary team. Although slightly different from 1 site to another, the multidisciplinary monitoring included nutritional support by a certified dietician (low-fat diet adapted to the caloric needs for both the mother and the fetus); regular TG measurements (at least once monthly during the first and second trimesters and bimonthly to biweekly during the third trimester if TG levels were >20 mmol/L); assessment of gestational diabetes (oral glucose tolerance test); evaluation of abdominal pain occurrence and fetal growth; and regular interactions between lipidologists, obstetricians, and nutritionists, among others.

### Pregnancies Outcomes

Among the 116 pregnancies reviewed, a total of 13 miscarriages and 9 fetal failures to thrive were observed. Both tended to be similarly distributed between the 2 groups (Table 2). However, a higher percentage of prematurity was observed in FCS than MCS (19.6% vs 6.2%, *P* = .05) (Table 2). Among premature deliveries, the mean number of gestational weeks before delivery was 33.7 ± 3.3 weeks. Labor was induced in 9 women (6 FCS and 3 MCS) who delivered prematurely. Overall, 59% of the labors were induced in FCS compared to 26% in MCS (*P* = .029) as illustrated in Table 2. There were 2 cases of fetal death following an AP crisis. Only 1 case of preeclampsia was reported in an MCS woman.

**Table 2.** Course of pregnancies in women with FCS and MCS

Variables	FCS (n = 20) (51 pregnancies)	MCS (n = 29) (65 pregnancies)	*P*-value
Miscarriages, n (%)	6 (11.8)	7 (10.7)	NS
Fetal failure to thrive, n (%)	3 (5.9)	6 (9.2)	NS
Prematurity, n (%)	10 (19.6)	4 (6.2)	.05
Induced labor, n (%)	30 (58.8)	17 (26.2)	.029
Delivery by cesarean section, n (%)	12 (23.5)	25 (38.5)	NS
Foetal birth weight (mean ± SD)	2.97 ± 0.67	3.29 ± 0.65	.018

Abbreviations: FCS, familial chylomicronemia syndrome (biallelic carriers and clinical FCS); MCS, multifactorial chylomicronemia syndrome (all subtypes); NS, nonsignificant (*P* > .1).

There was no difference in delivery by cesarean section between the 2 groups (32% in FCS and 48% in MCS, *P* = .14). However, FCS women delivered babies with a significantly lower weight (2.98 kg vs 3.29 kg, *P* = .018), which is consistent with the premature deliveries in this group.

## Discussion

This study reviewed a total of 116 pregnancies in a large cohort of women with chylomicronemia of different causes (2) followed in different lipid clinics. It highlights the serious risk of AP associated with severe or extreme HTG during pregnancy in the presence of chylomicronemia. Indeed, our results suggest that the frequency of AP during pregnancy could be at least 30-fold (MCS) to 120-fold (FCS) higher in women with chylomicronemia compared to the reported rate in the general population (0.35%) (12, 18, 19). However, a multidisciplinary follow-up during pregnancy for those who were previously diagnosed allowed a decrease in the occurrence of AP in FCS from 39% in the absence of monitoring to 7% (*P* = .01).

FCS (biallelic or clinical) is a rare and severe form of persistent chylomicronemia (1-10 per million), whereas MCS is, importantly, more frequent, with an estimated prevalence of 1/500 in North America (10, 25). It is estimated that 15% to 20% of subjects with MCS (1/10 000 individuals) are heterozygote carriers of FCS-causing variants (8). Despite the higher risk of AP in FCS compared to MCS, pregnancies in MCS might present a broader clinical challenge at the population level, considering its 1000-fold higher prevalence.

Compared to MCS, FCS women are usually diagnosed at a younger age, often during childhood, which should be associated with closer monitoring during pregnancies. Despite early diagnosis and closer follow-up, the proportion of AP occurring during pregnancy is significantly higher in FCS than in MCS (17% vs 5%, *P* = .02) (Fig. 2A), illustrating the high risk of AP associated with extreme HTG and severe persistent chylomicronemia. It has been previously reported that post-heparin LPL and hepatic lipase activities decrease during the second and the third trimesters of pregnancy (26), and it is well documented that plasma TG concentrations increase during the third trimester (14, 17), thus contributing to a greater AP risk in the presence of preexisting chylomicronemia or persistent lack of LPL bioavailability.

The occurrence of AP during pregnancy allowed the establishment of a diagnosis of FCS in 2 women and a diagnosis of MCS in 3 women who ignored their status and who never experienced an AP event before becoming pregnant. In FCS, 56% of AP occurred during the first pregnancy, whereas it did not occur in primigravida MCS women. This is consistent with the delayed expression of chylomicronemia and the greater contribution of age-dependent secondary factors affecting TG levels in MCS compared to FCS (10).

The percentage of miscarriages (11.8% vs 10.7%) and fetal failure to thrive (5.9% vs 9.2%) were not significantly different between the 2 cohorts (Table 2) and was similar to what is reported in the general population (8-15% and 8.6%, respectively) (27, 28), suggesting that chylomicronemia per se might not significantly influence fetal development. Compared to MCS, a higher proportion of FCS pregnancies were associated with prematurity (19.6% vs 6.2%, *P* = .05) (Table 2) or induced labor (59% vs 26%, *P* = .029) (Table 2). The reasons to induce labor and delivery were not always documented, but in most cases, it was a preventive measure to avoid an AP episode in the last weeks of pregnancy. Preeclampsia was not observed in FCS, and only 1 case occurred in an MCS woman during her second pregnancy. The prevalence of preeclampsia in this study was thus 0.9% (1/116), suggesting that chylomicronemia does not contribute to the risk of preeclampsia.

During pregnancy, particularly in the third trimester, estrogen concentration increases, which leads to higher VLDL synthesis by the hepatocytes (14). As a consequence, TG and VLDL cholesterol increase by 2.5-fold and low-density lipoprotein cholesterol by approximately 1.6-fold in the third trimester (17). In the general population, such increases rarely have significant clinical repercussions. The situation is different in the presence of sHTG when TG lipolysis is compromised due to a lower or complete lack of LPL bioavailability. The combination of LPL deficiency and increased TG-rich lipoproteins synthesis by the liver exacerbates the severity of the HTG phenotype, which can be life-threatening for both the mother and the fetus. When an AP episode occurs during pregnancy, the maternal mortality rate can reach 37%, whereas fetal lethality could be as high as 60% according to published data (29, 30). Several reports have previously highlighted the risk and morbidity of AP during pregnancy in the presence of sHTG or chylomicronemia (15, 31-37).

Currently, there are no clear guidelines for HTG management during pregnancy, but when TG levels are >4 mmol/L before pregnancy, the risk of AP should be considered (17). The significantly higher prevalence of AP in pregnant women with MCS (odds ratio: 30) compared to the general population and the resulting risk of AP might support a recommendation to systemically measure nonfasting TG levels before pregnancy and in the third trimester in women with a previous history of mild or moderate HTG.

Results of our study suggest that accurate monitoring and management of TG levels can substantially decrease the risk of AP in pregnant women with chylomicronemia of any cause. There were slight differences in the monitoring of pregnant women with FCS and MCS between sites. In particular, MCT oil was not part of the treatment plan for FCS women in France, was not systematically used in Canada, and, as illustrated in Table 1, was not used in MCS. However, multidisciplinary follow-up during the pregnancy remains essential to decrease chylomicronemia-associated risks. The team should ideally include obstetricians, lipidologists, endocrinologists, specialized nurses, and dieticians (17, 38).

Several case reports of successful pregnancies with or without AP using different treatments have been published (31-34, 36, 38-40). In this study, only 1 patient was treated with a fibrate and very few with omega-3 (Table 1). Most currently available TG-lowering agents are LPL-dependent and poorly effective when LPL activity is severely hampered, which is the case in FCS and a subgroup of MCS with persistent chylomicronemia (2, 3, 8). In addition, due to their poorly documented safety profile on the fetus, lipid-lowering agents are usually ceased during pregnancy. However, omega-3 fatty acids and fibrates (gemfibrozil, fenofibrate) have been used with mitigated success on top of an adapted diet in pregnant women with sHTG at very high risk of pancreatitis (1, 17, 41, 42). Some FCS pregnancies have required preventive hospitalizations during the last trimester. Parenteral nutrition and insulin infusion have been used with variable efficacy (15, 17, 38, 39). Plasmapheresis can also be considered (36, 40), but this highly specialized technique is not available everywhere, is invasive, and does not necessarily avoid AP episodes during pregnancy. Recently, it has been reported that plasmapheresis poorly contributes to health benefits and does not necessarily improve outcomes in patients at high risk of HTG-induced AP (43).

In recent years, new TG-lowering agents with a long duration of action have been developed to manage sHTG and chylomicronemia. These include LPL gene replacement therapy, ANGPTL3 or APOC3 small interfering RNA (siRNA) or antisense oligonucleotides, and gene or base editing strategies (44). Despite the lack of clinical data regarding the safety of these therapies in pregnant women, examples of successful pregnancies have been reported, opening the door to the possible use of new therapeutic agents with a long duration of action. In our study, 1 FCS woman became pregnant 8 weeks after having received plozasiran, an APOC3 siRNA, during a phase 3 clinical trial (45). The pregnancy went well, and she delivered a healthy baby. siRNA are agents have a short half-life in the bloodstream but a long duration of action (several months) given the long half-life in the hepatocytes. Another patient, who was not included in the present study due to a lack of data, was previously treated with a single intramuscular dose of Glybera, an adeno-associated virus-based *LPL* gene replacement therapy. She became pregnant approximately 1 year after the treatment administration. Her TG levels did not increase during the pregnancy; she had no significant complications and delivered a healthy baby (46). In 2024, Wanninayake et al (47) reported the cases of 2 pregnant FCS women using volanesorsen, an antisense oligonucleotide targeting APOC3 associated with TG reductions from 77% to 86% in FCS (48, 49). In the first case, the patient was using volanesorsen for 3 years when she found out (at 38 weeks) that she was pregnant. The treatment was stopped, and she delivered a healthy baby at 39 weeks of gestation, without any episode of AP during pregnancy. Another woman stopped volanesorsen 6 months before conception. Despite treatment with fibrates, TG levels increased, and she experienced an episode of AP at week 22. At week 23, volanesorsen was reintroduced in conjunction with plasmapheresis. She delivered a healthy baby at 35 weeks of gestation without any other AP episode (47).

The results reported herein derive from the analysis of a large multicentric cohort of pregnant women with chylomicronemia, combining FCS and MCS individuals with genetic susceptibility. This study describes major outcomes of pregnancies in the presence of extreme HTG, including miscarriages, fetal growth, prematurity, induced labor, and deliveries. The results highlight the high risk of AP events during pregnancy in the presence of chylomicronemia of different causes and the importance of early diagnosis and close monitoring. However, our study has limitations. Detailed data on the follow-up of patients before and during pregnancies, including compliance with the diet and the course of AP episodes when they occurred, were not always documented. In particular, data on AP severity, duration of hospitalizations, or organ failures were lacking. Our results must also be replicated in other cohorts from different parts of the world where access to accurate clinical or genetic diagnosis varies and where nutritional and environmental exposures are diverse.

It is crucial to gather information on access in communities where multidisciplinary care and resources are sparse, such as in remote regions or low- and middle-income countries. Because chylomicronemia during pregnancy is a high-risk condition and an unmet medical need, it would be clinically relevant to establish an international collaborative effort to garner information on the natural history of the disease, including the course of pregnancies, in order to facilitate equitable access to accurate diagnosis, follow-up, and treatment.

## Conclusion

Pregnancy importantly increases the risk of AP in women with chylomicronemia. The risk, proportional to the severity of the disease, is the highest in FCS although also substantially elevated in MCS compared to the general population. The occurrence of AP can be significantly decreased with an early diagnosis that could facilitate the monitoring and management of TG concentrations throughout the pregnancy by a specialized multidisciplinary team. Emerging TG-lowering agents that have a long duration of action, particularly APOC3 inhibitors, are progressively becoming available and efficiently decrease TG levels, even in patients lacking LPL bioavailability and presenting with recurrent or persistent chylomicronemia. More data are crucially needed to assess their safety and efficacy during pregnancy.

## Data Availability

The data underlying this article will be shared on reasonable request to the corresponding author.
